# Unraveling the Experience of Affection Across Marital and Friendship Interactions

**DOI:** 10.1007/s42761-024-00277-7

**Published:** 2024-09-25

**Authors:** Tabea Meier, Malena Otero, Simon X. Su, Jacquelyn E. Stephens, Chen-Wei Yu, Claudia M. Haase

**Affiliations:** 1 School of Education and Social Policy, Northwestern University, Evanston, IL USA; 2 Department of Psychology, University of Zurich, Zurich, Switzerland; 3 Healthy Longevity Center, University of Zurich, Zurich, Switzerland; 4 Mather Institute On Aging, Evanston, IL USA; 5 (by courtesy) Department of Psychology, Northwestern University, Evanston, IL USA

**Keywords:** Positive emotions, Affection, Dyadic interactions, Romantic relationships, Friendship

## Abstract

Feelings of affection (i.e., fondness and intense positive regard) are a hallmark of close relationships. Existing studies have often examined affection as a trait or behavior, but rarely as an emotional *experience* in social interactions. In two dyadic observational interaction studies (total *N* = 314 individuals), 49 US-based married couples (age range: 21–65) and 108 friendship dyads (age range: 15–26) engaged in two naturalistic 10-min conversations about (a) a topic of disagreement (conflict conversation) and (b) something they enjoyed doing together (pleasant conversation) and reported on their subjective emotional experiences (e.g., affection, using an emotion checklist following each conversation) and relationship satisfaction. Conversation transcripts were analyzed using natural language analysis. Results showed that experiences of affection were most strongly associated with experiences of compassion, amusement, and excitement, as well as with less anger, and were unrelated to most other negative emotions. Natural language analysis further showed that greater positive (but not negative) emotional tone was associated with greater affection (especially among friends in the pleasant conversations). Levels of affection were consistently higher in pleasant versus conflict conversations and similar across marital versus friendship interactions. Finally, experiences of affection were associated with greater relationship satisfaction for friends across conversations and for spouses (women) in the pleasant conversation. These findings contribute to our understanding of affection as a foundational emotional experience in close relationships and highlight avenues for future research.

**Supplementary Information:**

The online version contains supplementary material available at 10.1007/s42761-024-00277-7.

Affection is an intrinsically rewarding positive emotion in close relationships (Floyd, 2002; Fredrickson, 2016). People can experience affection—feelings of “fondness and intense positive regard” (Floyd, 2014, p. 3)—towards children, romantic partners, friends, pets, and others. Although affection has long interested laypeople, artists, philosophers, and psychologists, few studies have examined its experience in social interactions. The present investigation combined two observational interaction studies with married couples and friendship dyads to examine experiences of affection and (1) associations with positive and negative emotional experiences and linguistic markers of emotional tone, (2) differences across conversation and relationship types, and (3) links with relationship satisfaction.

## The Experience of Affection

Affection is central to many models of affiliation, attachment, and love (Bowlby, 2005; Depue & Morrone-Strupinsky, 2005; Floyd, 2002; Fredrickson, 2016). While affection shares similarities with other positive emotions, it is also conceptually and empirically distinct (Coan et al., 2006; Shiota et al., 2017), consistent with views of emotions as categories with “fuzzy boundaries” (Cowen & Keltner, 2017). Compassion and gratitude, for example, also promote care but typically arise when witnessing suffering or being benefitted (Stellar et al., 2017).

Research has often examined affection as a trait (Floyd, 2002; Pauley et al., 2014) or behavior (e.g., Bowlby, 2005; Carstensen et al., 1995; Coan et al., 2006; Debrot et al., 2013; Verstaen et al., 2020; see “Supplemental Materials” for more details), highlighting, for example, how affection behavior is important and quite different from other positive emotional behaviors in dyadic interactions (Carstensen et al., 1995; Verstaen et al., 2020). However, few studies have studied affection as an emotional *experience*.

Subjective emotional experiences play a key role in theories of human emotion (e.g., Barrett et al., 2007) and recent years have seen impressive advances in mapping human emotional experiences. For example, a large-scale study (Cowen & Keltner, 2017) found 27 distinct emotional experiences evoked by film clips, but affection was largely absent. Another large-scale study of daily emotional experiences (Chung et al., 2022) revealed a broader “love” emotion family, including affection, compassion, and gratitude. While insightful, these studies relied on solitary paradigms (e.g., film clip viewing, individual experience sampling) with little connection to contexts where affection naturally occurs—in interaction with close others.

In sum, it is unclear how the experience of affection in social interactions relates to other positive (e.g., compassion, excitement) and negative (e.g., anger, fear) emotional experiences and other markers of emotional functioning (e.g., emotional language during conversations, e.g., Meier et al., 2024a).

## In What Contexts Do People Experience Affection?

Romantic relationships and marriages are often seen as the realm of affection in adulthood (e.g., Finkel et al., 2015). Despite declining marriage rates, most individuals marry at least once (e.g., US Census Bureau, 2022).

However, affection also emerges in other close relationships (Bowlby, 2005) and many cultures view feelings of positive regard as foundational for both marriages and friendships (Floyd & Voloudakis, 1999). Marriages and friendships share many commonalities—both can provide intimacy (Shelton et al., 2010), interdependence (Kelley, 1983), and support (Gable & Bedrov, 2022)—but they are clearly also different. Notably, affection (especially if charged romantically) may be viewed as less appropriate (Floyd & Voloudakis, 1999) in friendships than marriages. 

Affection is moreover target-specific, felt towards a specific person (Floyd, 2002), and can fluctuate even with the same partner (Bhargava, 2023), consistent with dynamic views of emotions as adaptive responses to changing demands (Levenson, 1999). Positive interaction contexts may present special opportunities for pair-bonding (e.g., Fredrickson, 2016) and make the experience of affection more likely, although partners can experience affection also during conflict (Carstensen et al., 1995; Coan & Gottman, 2007).

In sum, it is unclear what contexts are most evocative of affection. How do macro (marriages versus friendships) and micro (pleasant versus conflict interactions) contexts shape the experience of affection?

## How is Affection Associated With Relationship Satisfaction?

Affection is often seen as the glue holding relationships together. Individuals with greater dispositional affection engage in more relationship-maintenance behaviors (Pauley et al., 2014) and are more likely to be in long-term romantic relationships (Floyd, 2002). Affection behaviors (e.g., handholding) can attenuate threat responses (Coan et al., 2006), foster closeness and positive affect (Debrot et al., 2013; Kolodziejczak et al., 2022), and are associated with higher relationship satisfaction (Muise et al., 2014).

Thus, affection at the trait and behavioral level appears to be linked to higher relationship satisfaction. But what about subjective experiences of affection that are not necessarily manifest in behavior – do they show similar links with relationship satisfaction?

## The Present Investigation

We examined subjective experiences of affection across two observational studies with married couples (aged 21–65) and friendship dyads (aged 15–26) from diverse socioeconomic backgrounds who engaged in conflict and pleasant conversations and reported on their emotional experiences and relationship satisfaction. To enhance ecological validity, this investigation focused on developmental stages where marriages and friendships are particularly salient. Marriage is among the most important relationships for many adults (Levenson et al., 2014). Friendships are important throughout the lifespan but are especially central for well-being during adolescence and emerging adulthood (Collins & Steinberg, 2006; Kroencke et al., 2023; Schwartz-Mette et al., 2020).

Analyses addressed three research questions. First, we explored associations of affection with other positive and negative emotional experiences and linguistic markers of emotional tone (Boyd et al., 2022). Second, we examined affection across (a) marriages versus friendships and (b) pleasant versus conflict conversations, expecting greater affection in marriages (versus friendships) and pleasant (versus conflict) conversations. Third, we examined links between affection and relationship satisfaction, expecting positive actor associations and exploring partner associations (associations with own versus partner’s satisfaction; Kenny et al., 2006). For (2) and (3), analyses controlled for age (Carstensen et al., 1995), gender (Muise et al., 2014), socioeconomic status ( Hittner & Haase, 2021), and (for friendship pairs) dyad type (same- versus mixed-gender; Floyd & Morman, 1997). For (3), additional follow-up analyses controlled for other positive and negative emotional experiences.

## Method

We report how we determined our sample sizes, all data exclusions, all manipulations, and all measures in the study. Findings from the larger research projects have been published (Hittner & Haase, 2021; Hittner et al., 2019; Meier et al., 2024a) but do not overlap with the current investigation.

## Participants

### Marriage Study

Data for this study came from a larger research project on socioemotional functioning in married spouses who had at least one child aged 5 to 18 years from the greater Chicago area, IL, USA. Participating couples were recruited through advertisements on public transportation, flyers, and online postings. We examined a sample of 49 couples (50% female; age: *M* = 42.89, *SD* = 8.65 years, range: 21–65) who completed laboratory-based interaction tasks and provided complete observations for the affection and relationship satisfaction measures. We excluded couples (*n* = 7) with incomplete affection or relationship satisfaction data. Spouses with missing data did not differ from others in terms of sociodemographic characteristics (age, years of education, income), affection and relationship satisfaction (*p*s ≥ 0.082). The demographics of the final sample were as follows: Income [annual household income before taxes]: less than $20,000: 14.3%, $20,001—$35,000: 12.2%, $35,001—$50,000: 14.3%, $50,001—$75,000: 12.2%, $75,001—$100,000: 14.3%, $100,001—$150,000: 20.4%, and greater than $150,000: 12.2%); Education [in years]: *M* = 15.95, *SD* = 2.64, range: 8–21); 44.9% were white, 36.7% African American/Black, 6.1% Latinx/Hispanic, 7.1% Asian/South Asian, 1.0% Hawaiian/Pacific Islander and 4.1% Multiracial. On average, spouses had been married for *M* = 11.69 years (*SD* = 6.63, range: less than 1 year – 28 years) at the time of the study. Couples were compensated with $100.

### Friendship Study

Data for this study came from a larger online-based research project on socioemotional functioning in friendship dyads from the US. Participating friends were recruited through flyers and online postings. We examined a sample of 108 friendship pairs (58.8% female, 38.4% male, 2.8% non-binary/diverse; age: *M* = 19.98, *SD* = 2.22 years, range: 15–26) who completed online interaction tasks and provided complete observations for the affection and relationship satisfaction measures. We excluded friend pairs (*n* = 2) with incomplete affection or relationship satisfaction data. Friends with missing data did not differ from other friends in terms of sociodemographic characteristics (age, paternal education and friendship satisfaction (*p*s ≥ .126), but participants with missing data had fewer years of maternal education (*p* = 0.035), lower family income (*p* < .001), and reported less affection in the conflict conversation (*p* < .001). The demographics of the final sample were as follows: Income [annual personal household income before taxes]: less than $20,000: 73.1%, $20,001—$35,000: 9.3%, $35,001—$50,000: 4.6%, $50,001—$75,000: 4.6%, $75,001—$100,000: 3.2%, $100,001—$150,000; 1.9%, and greater than $150,000: 1.4%); Education [in years]: *M* = 13.19, *SD* = 2.22, range: 8–18); 39.4% were white, 14.4% African American/Black, 7.4% Latinx/Hispanic, 29.6% Asian/South Asian, and 9.3% Multiracial. On average, friends had been in their current friendship for *M* = 5.65 years (*SD* = 4.36, range: less than 1 year – 19 years) at the time of the study. Each friend was compensated with $20.

## Statistical Power Considerations

We conducted several sensitivity analyses using GPower (Faul et al., 2007), accounting for the different analytical approaches used across research questions (i.e., dyadic mixed models, t-tests).

Regarding differences in affection across conversations as well as links with relationship satisfaction, we conducted a sensitivity analysis reflecting the dyadic nature of the analyses. Guided by other work in this area (e.g., Gordon et al., 2022), we calculated the effective sample size based on the design effect formula, *N*_effective_ = *N*/(1 + (*n*_cluster_ – 1) × ρ), which included the intraclass correlation coefficient (ICC or ρ) to account for non-independence within dyads.

### Affection Across Conversations

With a cluster size = 4 (two partners, two conversations) and an ICC = .16 (Marriage Study) and an ICC = .32 (Friendship Study), this revealed an effective sample size of 132.43 (Marriage Study) and 220.41(Friendship Study), respectively. Sensitivity analyses revealed that at a statistical power of 0.80 and an alpha level of 0.05, the Marriage Study was sufficiently powered to detect effects of ρ (population correlation coefficient) = .24 or larger, and thus small-to-medium effects (per guidelines by Cohen, 1992); the Friendship Study was sufficiently powered to detect effects of ρ (population correlation coefficient) = .19 or larger, and thus small-to-medium effects (per guidelines by Cohen, 1992).

### Affection Across Relationship Types

Regarding the comparison of affection across relationship types (i.e., married versus friendship dyads), a sensitivity analysis for two-sample t-tests revealed that at a statistical power of .80, an alpha level of .05 and group sizes of *N* = 98 (Marriage Study) and *N* = 216 (Friendship Study) individuals, our samples were sufficiently powered to detect differences of d = 0.34 or larger, and thus small-to-medium-sized effects (per guidelines by (Cohen, 1992).

### Affection and Relationship Satisfaction

With a cluster size = 2 (two partners) and an ICC = .14 (Marriage Study) and an ICC = .20 (Friendship Study),^1^ this revealed an effective sample size of 85.96 (Marriage Study) and 180 (Friendship Study), respectively. Sensitivity analyses revealed that at a statistical power of .80 and an alpha level of .05, the Marriage Study was sufficiently powered to detect effects of ρ (population correlation coefficient) = .29 or larger, and thus medium-sized effects (per guidelines by Cohen, 1992); the Friendship Study was sufficiently powered to detect effects of ρ (population correlation coefficient) = .21 or larger, and thus small-to-medium effects (per guidelines by Cohen, 1992).

## Procedure

Dyads were invited to a laboratory-based (Marriage Study) or online (Friendship Study) assessment of socioemotional functioning and engaged in several videotaped dyadic interactions, following established procedures (see e.g., Gottman & Levenson, 1992). The present investigation focused on (a) a 10-min conflict conversation about a topic of disagreement, and (b) a 10-min pleasant conversation about something the dyad enjoys doing together (with the order of the conflict and pleasant conversation counterbalanced in the Marriage Study). Each participant independently completed emotion checklists after each conversation and, at the end of the study, a series of questionnaires assessing sociodemographic information and relationship satisfaction, among other measures not relevant to the present investigation. Both studies were approved by the Northwestern Institutional Review Board.

## Measures

### Subjective Emotional Experiences

After each conversation, participants were asked to “Please indicate how strongly [1 = not at all; 9 = strongest ever felt] you felt each emotion during the conversation you just had” and reported on their positive (e.g., affection, amusement, calm, compassion, excitement, pride) and negative (e.g., anger, disgust, embarrassment, fear, sadness, shame) emotional experiences. We focused on positive and negative emotional experiences that were assessed in both studies to ensure parallel analysis.

### Conversations’ Emotional Tone

All conversations were transcribed and analyzed with the Linguistic Inquiry and Word Count (LIWC-22; Boyd et al., 2022) to quantify the rate of positive and negative emotion words used (as relative % of all words used in a given conversation) as indicators of positive and negative emotional tone, respectively (Meier et al., 2024a). We relied on the LIWC-22 categories “tone_pos”, “tone_neg” (Boyd et al., 2022).

### Relationship Satisfaction

Relationship satisfaction was assessed in terms of marital satisfaction and friendship satisfaction, respectively. In the Marriage Study, spouses completed the 15-item Locke-Wallace Short Marital-Adjustment Test (Locke & Wallace, 1959). Example items are: “Do you ever wish you had not married? [1 = frequently, 4 = never]’ or “Do you confide in your mate? [1 = almost never, 4 = in everything]”, and the standard weight methods were used for scoring (see Locke & Wallace, 1959). On average, spouses were moderately satisfied with their relationship (wives: *M* = 92.61, *SD* = 26.95; husbands: *M* = 90.67, *SD* = 25.35; possible range: 2–158, and the measure showed good internal consistency (α = .80).

In the Friendship Study, friends completed the 16-item McGill Friendship Questionnaire (MFQ-RA; Mendelson & Aboud, 1999). Example items are: “I am happy with my friendship with ______. [-4 = very much disagree, 4 = very much agree]” or “I am glad that ______ is my friend. [-4 = very much disagree, 4 = very much agree]”. We focused on the satisfaction subscale of the scale. On average, friends were highly satisfied with their friendship (*M* = 3.45, *SD* = 0.87; possible range: -4 to 4), and the measure showed good internal consistency (α = .93).

### Covariates

Covariates included age, gender, and socioeconomic status (SES). In the Marriage Study, which consisted of mixed-sex couples, there was one effect-coded gender variable (1 = husband, -1 = wife). In the Friendship Study, which consisted of same- and mixed-sex dyads and included more diverse gender identities, there were two effect-coded gender variables: male-versus-female (1 = male, 0 = diverse, -1 = female) and male-versus-diverse (1 = male, 0 = female, -1 = diverse), and we also included dyad type (1 = same-gender, -1 = mixed-gender) as a covariate. SES was measured using a standardized composite score of education (“What is the highest level of education you have obtained?”; 8 years = high school/GED, 21 years = PhD., MD, or other professional degree) and household income (“What is your family’s annual household income before taxes?”; 1 = less than $20,000, 7 = greater than $150,000; see e.g., (Hittner & Haase, 2021). For the Friendship Study, a composite of family household income, maternal, and paternal education was used instead. Results were stable when repeating the analyses using a personal SES composite (based on personal household income and own education).

## Data Analyses

Analyses were conducted separately for the Marriage and Friendship study. Dyads from the former were treated as distinguishable based on gender (see Kenny et al., 2006). Dyads from the latter, which contained both same-gender and mixed-gender dyads, were treated as indistinguishable, in line with state-of-the-art procedures in dyadic data analyses (Kenny et al., 2006; West, 2013). Preliminary analyses examined descriptive statistics (i.e., affection, emotional tone of the conversations).

### Affection and Other Emotional Experiences

Correlation analyses were used to explore associations between affection and other positive and negative emotional experiences across conflict and pleasant conversations. We characterized effect sizes using conventional guidelines (Cohen, 1992; small: *r* = .10, medium: *r* = .30, large: *r* = .50). Mixed models were used to explore associations between affection and emotional tone.

### Affection Across Conversations and Relationship Types

Linear mixed models in SPSS and SAS were used to examine differences in affection across conversations (1 = conflict, -1 = pleasant) treating both dyad members and conversation types (conflict, pleasant) as repeated measures (resulting in four observations per dyad). This procedure follows recommendations by West (2013) for analysis of data with a two-level crossed structure (individuals nested in dyads; both individuals participated in the same conversations). Separate error variances were calculated and allowed to correlate to account for interdependence within dyads and conversations (Kenny et al., 2006; West, 2013). Analyses were controlled for age, gender, SES and (for the Friendship Study) dyad type (1 = same-gender, -1 = mixed-gender). T-tests were used to explore mean-level differences in affection between marriages and friendships, separately for conflict and pleasant conversations, and additional Bayesian Equivalence tests, assuming a uniform distribution and the full range of possible differences (0–8; based on our 9-point affection scale) due to lack of prior evidence in this area, were used to strengthen the conclusions.

### Affection and Relationship Satisfaction

Actor-Partner-Interdependence models (APIM; Kenny et al., 2006) were computed using the package dyadr in R (Garcia & Kenny, 2020) to investigate associations between affection and relationship satisfaction. APIM allows for the simultaneous estimation of actor (e.g., between own affection and marital satisfaction) and partner associations (e.g., between own affection and the partner’s marital satisfaction) while accounting for dyadic interdependence (Kenny et al., 2006; West, 2013). For distinguishable dyads (Marriage Study), separate error variances were estimated for husbands and wives (Garcia & Kenny, 2020; Kenny et al., 2006; West, 2013).

We computed separate APIMs for each conversation (conflict, pleasant) and for each sample (Marriage, Friendship) with affection as the independent variables and relationship satisfaction as the dependent variables. Analyses were controlled for age, gender, socioeconomic status, and (for the Friendship Study) dyad type (same- versus mixed-gender). For distinguishable dyads (Marriage Study), interaction terms between affection*gender were also included in the main analyses, since gender was the distinguishable feature of this sample (see Kenny et al., 2006). Follow-up analyses tested associations between affection and relationship satisfaction for (1) specificity (when controlling for other positive and negative emotions, using composite variables averaging across all positive emotions except affection and all negative emotion, respectively) and (2) generalizability across different levels of socioeconomic status (by including the interaction terms between affection and SES). The results did not change when using separate interaction terms for income and education instead of the SES composite; hence, we report the results based on the SES composite (see Emery & Finkel, 2022; Johnson et al., 2023 for considerations on different operationalizations of SES). Additionally, for the Friendship Study, which contained both same- and mixed-gender dyads, separate follow-up analyses tested for (3) generalizability across genders and dyad types (by including the interaction terms with affection).

## Results

### Preliminary Analyses

Means and standard deviations for experiences of affection were as follows: *M* = 4.84 (*SD* = 2.46) for spouses in the conflict conversation and *M* = 5.98 (*SD* = 2.06) for spouses in the pleasant conversation; *M* = 4.93 (*SD* = 2.30) for friends in the conflict conversation and *M* = 5.62 (*SD* = 2.21) for friends in the pleasant conversation (possible range: 1–9).

In the Marriage Study, about *M* = 1.92% (*SD* = 0.84) of the words spoken in the conflict conversation conveyed a positive emotional tone, and *M* = 1.06% (*SD* = 0.60) conveyed a negative emotional tone, which is comparable to other marital interaction studies (e.g., Han et al., 2021). Similarly, in the pleasant conversation, about *M* = 2.71% (*SD* = 1.04) of the words spoken conveyed a positive emotional tone and *M* = 0.72% (*SD* = 0.48) conveyed a negative emotional tone. Similarly, in the Friendship Study, about *M* = 2.01% (*SD* = 0.61) of the words spoken in the conflict conversation conveyed a positive emotional tone and *M* = 1.28% (*SD* = 0.59) conveyed a negative emotional tone. In the pleasant conversation, about *M* = 2.81% (*SD* = 0.96) of the words spoken conveyed a positive emotional tone and *M* = 0.90% (*SD* = 0.44) conveyed a negative emotional tone. Conversations between married spouses versus friendship pairs did not differ in terms of positive emotional tone (pleasant conversation: t(296) = -0.76, *p* = .450, conflict conversation: t(131.33) = -0.90, *p* = .368) but friends’ conversations scored higher on negative emotional tone than marital conversations (pleasant conversation: t(296) = -3.20, *p* = .002, conflict conversation: t(294) = -2.96, *p* = .003).

### Affection and Other Emotional Experiences

Overall, experiences of affection were most consistently associated with higher compassion, amusement, and excitement (see Tables 1 and 2). There were also some correlations between affection and calmness (except for married spouses in the pleasant conversation) and pride (especially for friends). Affection was unrelated to most negative emotions, with a few exceptions. For example, married spouses experienced less anger when experiencing greater affection (especially in the conflict conversation), and friends also experienced some sadness when experiencing greater affection in the conflict conversation. Correlations between affection and other positive and negative emotional experiences are shown in Table 1 (Marriage Study) and Table 2 (Friendship Study), correlations with other key study variables, means and standard deviations in Supplemental Online Material (Tables S1-S3).

**Table 1:**
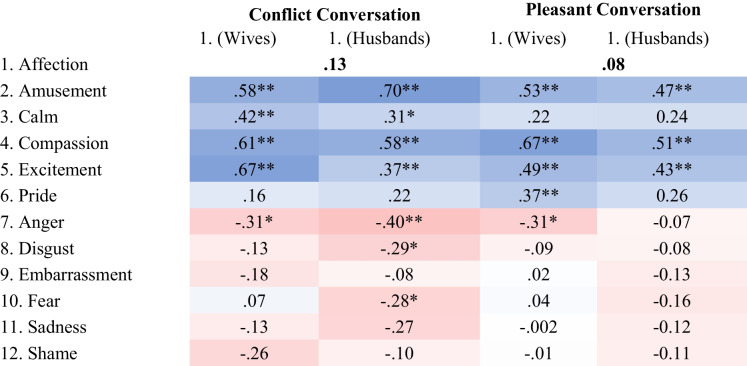
Marriage Study: Correlations Between Affection and other Positive and Negative Emotional Experiences

Correlations between husbands and wives are presented in bold. Values color coded from negative (red) to positive (blue). * *p* < .05, ** *p* < .01

**Table 2:**
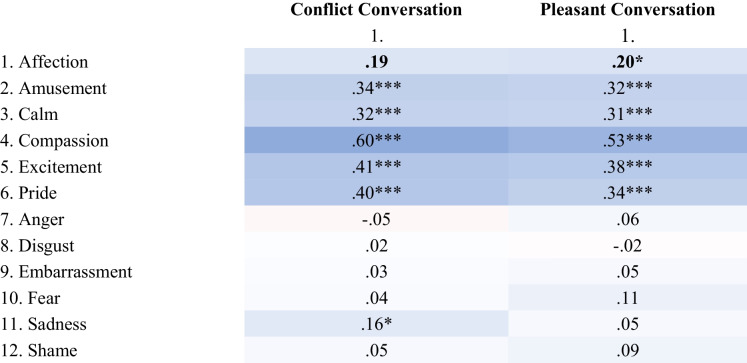
Friendship Study: Correlations Between Affection and other Positive and Negative Emotional Experiences

Presented are the overall within-partner correlations. The correlations between friends are presented in bold as intra-class correlations, according to recommendations by Griffin and Gonzalez, (1995) for indistinguishable dyads. P-values for pairwise intra-class correlations were adjusted for dependent observations in indistinguishable dyads (see Griffin & Gonzalez, 1995; O’Connor, 2019). Values color coded from negative (red) to positive (blue). * *p* < .05, ** *p* < .01, *** *p* < .001

#### Affection and Conversations’ Emotional Tone

Overall, experiences of affection were associated with positive (but not negative) emotional tone in the pleasant (but not conflict) conversations. Specifically, in friendship interactions, experiences of affection were linked to positive emotional tone in the pleasant (but not conflict, *p* = .074) conversation (B = 0.40, *SE*(B) = 0.17, Beta = 0.17, *p* = .022). In marital interactions, there was a similar trend for experiences of affection to be linked with positive emotional tone in the pleasant (but not conflict, *p* = .346) conversation, although below typical thresholds of statistical significance (B = 0.40, *SE*(B) = 0.21, Beta = 0.20, *p* = .068). None of the associations between experiences of affection and negative emotional tone were statistically significant (*p*s ≥ .091).

### Affection Across Conversations and Relationship Types

**Fig. 1:**
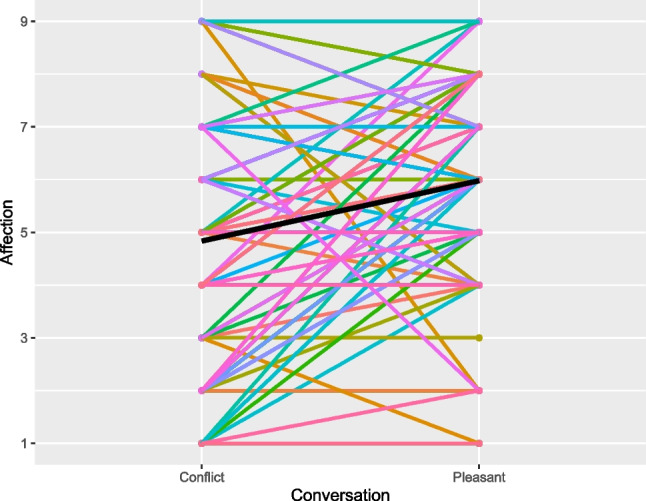
Marriage Study: Affection Across Conflict and Pleasant Conversations. *Note.* Affection was assessed on a scale ranging from 1 (= not at all) to 9 (= strongest ever felt). Members of the same dyad are presented in the same color. The black line represents the mean across participants

**Fig. 2:**
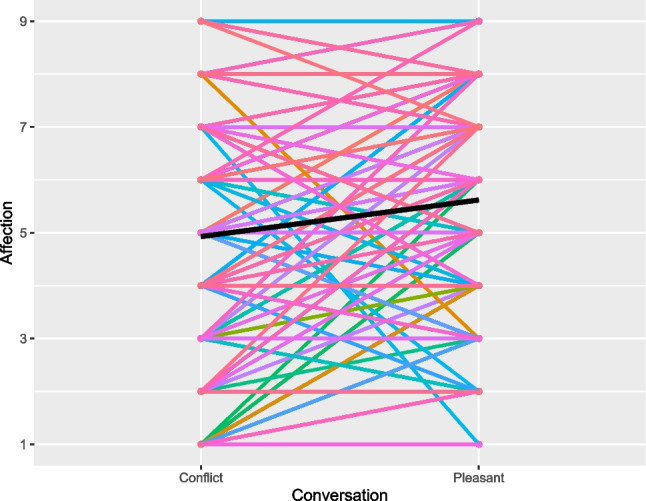
Friendship Study: Affection Across Conflict and Pleasant Conversations. *Note.* Affection was assessed on a scale ranging from 1 (= not at all) to 9 (= strongest ever felt). Members of the same dyad are presented in the same color. The black line represents the mean across participants

Overall, married spouses and friends experienced greater affection in the pleasant than in the conflict conversation and overall affection levels were similar across marriages and friendships. Specifically, on average, married spouses experienced greater affection in the pleasant than in the conflict conversation, indicated by a statistically significant main effect of conversation (B = -0.55, *SE*(B) = 0.13, *p* = .0001; see Fig. 1). The intraclass correlation of affection between the conflict and pleasant conversations was ICC = .36 for husbands, and ICC = .28 for wives, suggesting that affection varied across the two conversations, with small-to-moderate differences between the two conversations (Cohen’s d = 0.42 for husbands, Cohen’s d = 0.59 for wives). Similarly, friends experienced greater affection in the pleasant than in the conflict conversation (B = -0.35, *SE*(B) = 0.07, *p* < .0001; see Fig. 2). The intraclass correlation of affection between conflict and pleasant conversations was ICC = .70 for friends, suggesting rather high within-person stability in affection with relatively small differences between the two conversations (Cohen’s d = 0.31 for friends). Across marriages and friendships, there were no gender differences in affection indicated by non-significant main effects of gender (*p*s ≥ .357) and gender*conversation interactions (*p*s ≥ .403). Levels of affection were comparable across married spouses and friends, as indicated by non-significant two-sample t-tests for both the pleasant (*t*(312) = 1.36, *p* = .174) and conflict (*t*(312) = -0.31, *p* = .756) conversation (see Figs. 1 and 2, Table S1). This was further supported by Bayesian Equivalence Tests: In the pleasant conversation, with a mean difference = 0.36 and standard error of the difference (*SE*) = 0.26, this yielded a Bayes factor B_U[0,8]_ = 0.19, suggesting moderate relative evidence for no difference (Dienes, 2021). Similarly, in the conflict conversation, with a mean difference = -0.09 and standard error of the difference (*SE*) = 0.29, this yielded a Bayes factor B_U[0,8]_ = 0.04, suggesting strong relative evidence for no difference (Dienes, 2021).

### Affection and Relationship Satisfaction

Overall, experiences of affection were associated with greater relationship satisfaction for friends across conversations and for wives in the pleasant conversation (with some robustness when controlling for other positive emotions, including compassion). The full APIM results with all estimates are reported in Table 3 (Marriage Study) and Table 4 (Friendship Study).

**Table 3:** Affection and Relationship Satisfaction in the Marriage Study: Results From Actor-Partner Interdependence Models (APIM)

	Relationship Satisfaction			
	B [95%CI]	*SE*(B)	β	*p*-value
Pleasant Conversation				
Gender	8.11 [-3.75, 19.96]	6.05	-0.08	.184
Affection				
Actor effect	1.58 [-0.91, 4.06]	1.27	0.12	.218
Partner effect	2.46 [-0.03, 4.95]	1.27	0.19	.056
Affection				
Actor effect*gender	-3.07 [-5.68, -0.46]	1.33	-0.24	.024^*^
Partner effect*gender	1.37 [-1.24, 3.99]	1.33	0.11	.306
Conflict Conversation				
Gender	-0.24 [-9.57, 9.09]	4.76	-0.08	.960
Affection				
Actor effect	1.19 [-1.04, 3.43]	1.14	0.11	.297
Partner effect	1.45 [-0.77, 3.67]	1.13	0.14	.205
Affection				
Actor effect*gender	-1.42 [-3.88, 1.03]	1.25	-0.13	.260
Partner effect*gender	1.07 [-1.38, 3.51]	1.25	0.10	.395

B = unstandardized estimate, CI = confidence interval, *SE* = standard error of estimate, β = standardized estimate (based on the overall standard deviation across all participants). Gender (1 = husband, -1 = wife) was effect-coded. All models controlled for age and SES (all *p*s > .106). Interactions of gender with age and SES were non-significant and dropped from the models. * *p* < .050

**Table 4:** Affection and Relationship Satisfaction in the Friendship Study: Results From Actor-Partner Interdependence Models (APIM)

	Relationship Satisfaction			
	B [95%CI]	*SE*(B)	β	*p*-value
Pleasant Conversation				
Gender (male-versus-female)				
Actor effect	-0.004 [-0.28, 0.27]	0.14	-0.005	.976
Partner effect	0.15 [-0.12, 0.42]	0.14	0.17	.280
Gender (male-versus-diverse)				
Actor effect	-0.16 [-0.65, 0.32]	0.25	-0.19	.511
Partner effect	-0.06 [-0.54, 0.43]	0.25	-0.07	.816
Dyad type	-0.03 [-0.17, 0.11]	0.07	-0.04	.651
Affection				
Actor effect	0.06 [0.004, 0.11]	0.03	0.15	.036^*^
Partner effect	-0.02 [-0.08, 0.03]	0.03	-0.06	.417
Conflict Conversation				
Gender (male-versus-female)				
Actor effect	-0.01 [-0.28, 0.26]	0.14	-0.01	.938
Partner effect	0.17 [-0.10, 0.44]	0.14	0.20	.219
Gender (male-versus-diverse)				
Actor effect	-0.16 [-0.64, 0.33]	0.25	-0.18	.524
Partner effect	-0.09 [-0.58, 0.39]	0.25	-0.11	.709
Dyad type	-0.04 [-0.18, 0.10]	0.07	-0.04	.593
Affection				
Actor effect	0.06 [0.01, 0.11]	0.03	0.16	.019^*^
Partner effect	-0.03 [-0.08, 0.02]	0.03	-0.07	.316

B = unstandardized estimate, CI = confidence interval, *SE* = standard error of estimate, β = standardized estimate. Gender, male-versus-female (1 = male, 0 = diverse, -1 = female), male-versus-diverse (1 = male, 0 = female, -1 = diverse), and dyad type (1 = same gender, -1 = mixed gender) were effect-coded. All models controlled for age and SES (all *p*s > .191). * *p* < .050

#### Marriage Study

Specifically, in the marriage study, controlling for age, gender, and SES, there was a significant interaction between the actor’s affection and gender (B = -3.07, *SE*(B) = 1.33, Beta = -0.24, *p* = .024), suggesting that affection was linked with one’s own relationship satisfaction for wives (but not for husbands) in the pleasant conversation. This association was robust when controlling for all other positive emotions (B = -2.99, *SE*(B) = 1.33, Beta = -0.24, *p* = .027) and negative emotions (B = -3.06, *SE*(B) = 1.34, Beta = -0.24, *p* = .025).

There was also a marginally significant partner association (B = 2.46, *SE*(B) = 1.27, Beta = 0.19, *p* = .056) between affection in the pleasant conversation and relationship satisfaction. The association was not moderated by gender (*p* = .306) and was robust when controlling for all other positive emotions (B = 3.75, *SE*(B) = 1.70, Beta = 0.30, *p* = 0.030), but not negative emotions (*p* = .083). Associations between affection and relationship satisfaction were not moderated by SES (indicated by non-significant interaction effects, *p*s ≥ .469). In the conflict conversation, all actor and partner associations between affection and relationship satisfaction were non-significant (*p*s ≥ .205; see Table 3).

#### Friendship Study

In the friendship study, controlling for age, gender, SES, and dyad type (same- versus mixed-gender), there was a statistically significant actor association (B = 0.06, *SE*(B) = 0.03, Beta = 0.15, *p* = .036) between affection and relationship satisfaction in the pleasant conversation. This association was no longer statistically significant when controlling for all other positive emotions (*p* = 0.099), but was robust when controlling for negative emotions (B = 0.06, *SE*(B) = 0.03, Beta = 0.14*, p* = .016).

Similarly, the actor association between affection and relationship satisfaction was significant in the conflict conversation (B = 0.06, *SE*(B) = 0.03, Beta = 0.16, *p* = .019), and this association was robust when controlling for all other positive emotions (B = 0.07, *SE*(B) = 0.03, Beta = 0.17*, p* = .047) and for negative emotions (B = 0.07, *SE*(B) = 0.03, Beta = 0.18*, p* = .009). Actor associations between affection and relationship satisfaction were not moderated by SES, gender, or dyad type, indicated by non-significant interaction terms (*p*s ≥ .425). The pseudo R^2^ (based on the multiple correlation squared) was R^2^ = .018 for the pleasant conversation, and R^2^ = .024 for the conflict conversation, suggesting that friends’ affection in both conversations explained similar amounts of variance (around 2%) in relationship satisfaction. None of the partner associations between affection and relationship satisfaction were statistically significant (*p*s ≥ .316) in either the conflict or pleasant conversation.

## Discussion

This investigation responds to calls for more attention to positive processes in affective and relationship science (e.g., Algoe, 2019; Shiota et al., 2017) by examining a key positive emotional experience in close relationships—affection. Across two dyadic observational studies with married and friendship pairs, findings showed that experiences of affection were (1) related to, yet distinct, from other positive emotions and largely unrelated to negative emotions; (2) consistently higher in pleasant than in conflict conversations but similar across marital versus friendship interactions; and (3) were linked with one’s own relationship satisfaction in some contexts (i.e., for friends in conflict and pleasant conversations, for wives in the pleasant conversation).

### The Experience of Affection in Affective Space

Across marriages and friendships, affection was associated with self- and other-oriented positive emotional experiences, consistent with a relative blending of positive emotional states (Cowen & Keltner, 2017; Fredrickson, 1998). Specifically, affection was linked with compassion – aligning with an emotion family of “love” (Chung et al., 2022); amusement – underscoring the importance of humor and playfulness in close relationships (Brauer et al., 2021; Horn et al., 2019); and excitement – hinting at the role of positive activation in pair bonding (Aron et al., 2000). Affection showed few associations with negative emotions except for moderate, negative correlations with anger. Similarly, our language findings showed that experiences of affection were associated with positive (but not negative) emotional tone of the conversations, particularly in pleasant conversations among friends. These findings provide some of the first evidence on affection’s location in the emotional experience space in dyadic interactions (Chung et al., 2022; Cowen & Keltner, 2017) and linguistic correlates (Floyd, 2002). They expand upon previous work (Gonzaga et al., 2001) by suggesting that positive interpersonal emotional experiences are not merely about reduced negative emotions.

### Affection in Relationship Contexts

Trait views see affection as a stable disposition (e.g., Floyd, 2002) and “lack of loving feelings” is among the most difficult problems in couple therapy (Whisman et al., 1997). Our findings showed variability in feelings of affection, depending on conversational context. Spouses and friends experienced greater affection when discussing enjoyable activities, but, on average, still experienced substantial affection (just below the midpoint of the scale) when discussing disagreements. Capitalizing on positive experiences is an important driver of connection in relationships (Gable & Reis, 2010) and seems to also promote feelings of affection.

While affection appeared to be context-dependent at the micro (conversational) level, few differences emerged at the macro (relationship type) level. In the present study, middle-aged spouses and adolescent/young adult friends experienced similar levels of affection. While it remains to be seen whether this finding generalizes across other developmental stages (e.g., friendships in later adulthood), it suggests affection is not unique to romantic relationships, converging with other research (Shelton et al., 2010) and criticisms of couple-centric perspectives (DePaulo, 2023). This is notable in light of ever-increasing demands on modern marriages (Finkel et al., 2015).

### Affection and Relationship Satisfaction

Experiences of affection were associated with greater relationship satisfaction, especially among friends. Affection was linked with greater relationship satisfaction across conflict and pleasant conversations for friends, and in pleasant conversations for wives (but not husbands). These findings converge with prior work on affectionate behavior (Debrot et al., 2013; Muise et al., 2014) and positive emotions (Fredrickson, 2016; Gottman & Gottman, 2017) and suggest considerable specificity (associations for friends in conflict and for wives in pleasant conversations remained significant, even when controlling for other positive and negative emotional experiences). These findings also indicate that the experience of affection may be one, though not sole, characteristic of happy relationships. In fact, prior work (Verstaen et al., 2020) indicates that affection may reflect passionate love, which is central initially but may diminish as love evolves and companionate love becomes more salient (Hatfield & Walster, 1985). While findings from our sample of established marriages speak to this idea, future research should follow up on this.

### Strengths, Limitations, and Future Directions

The present investigation has several strengths, including its focus on (a) both romantic and friendship dyads, (b) conflict and pleasant conversations, (c) dyadic design, and (d) consideration of confounding variables (age, gender, socioeconomic status, dyad type). The study also has limitations. Our samples were (a) convenience samples and (b) small compared to large survey-based studies and some other dyadic interaction studies (e.g., Carstensen et al., 1995). Moreover, although the samples were notable in their inclusion of participants from highly diverse socioeconomic, racial and ethnic backgrounds, which is rare in relationship research (McGorray et al., 2023), the married sample (c) consisted of mixed-gender couples due to local marriage legislation when data was collected. Affection was assessed (d) with a single item, and studies were (e) cross-sectional, US-based, and examined spouses and friends at different developmental stages.

Future research should probe generalizability across different gender orientations, cultural contexts, relationship qualities (see Barton et al., 2020; Jakubiak, 2022). Studies comparing romantic relationships and friendships within the same individuals are needed to disentangle effects of relationship type and age. Finally, future studies might investigate continuous experiences of affection (using rating dials) and associations with affectionate behaviors (Wu et al., 2021), passionate and companionate love (Verstaen et al., 2020), and responsiveness (Reis & Gable, 2015).

## Conclusion

Close relationships are hotbeds of emotion (Levenson et al., 2014; Meier et al., 2024b) and these emotions are important predictors of relationship functioning, well-being, and health (Carstensen et al., 1995; Haase et al., 2016; Wells et al., 2022). Traditionally focused on negative emotions, affective and relationship sciences have begun to reveal important insights into positive emotions (e.g., awe, compassion; Algoe, 2019; Shiota et al., 2017). We hope that the present studies spur interest in feelings of affection as foundational positive emotional experiences in relation with close others.

## Supplementary Information

Below is the link to the electronic supplementary material.

Supplementary file1 (DOCX 51.4 KB)

## Footnotes

1 Analyses were performed separately for conflict and pleasant conversations. For sensitivity analyses, we conservatively used the higher ICC for the Marriage and Friendship Study, respectively.

## Additional Information

### Data Availability Statement

The datasets from the present studies are not publicly available due to elevated concerns about participant re-identifiability and sensitive data in dyadic studies outlined by Eli Finkel (see Joel et al., 2018). Materials and data from this manuscript will be shared upon request with other researchers. Analysis scripts: The analysis scripts are publicly available on the Open Science Framework: https://osf.io/m7pb5/.
