# Supplementary material for: Unraveling the Experience of Affection Across Marital and Friendship Interactions
Source: Affect Sci. 2024 Sep 25;6(1):104–16. doi: 10.1007/s42761-024-00277-7 (PMC11904024; doi:10.1007/s42761-024-00277-7)
Supplement: Supplementary file 1 — Supplementary file1 (DOCX 51.4 KB) [file 42761_2024_277_MOESM1_ESM.docx]

Supplemental Online Material (SOM) for:

# Unraveling the Experience of Affection Across Marital and Friendship Interactions

**Prior Insights from Studies on Affectionate Behaviors**

Thus far, affection has predominantly been studied as a behavior and linked to a host of benefits for individual and relational well-being (see Jakubiak & Feeney, 2017 for a review). Expressions of affection (e.g., hugs, touch) can buffer against stress, as shown by daily diary (e.g., Debrot et al., 2014) and experimental studies (e.g., Ditzen et al., 2007). Affectionate touch has been linked to lower stress reactivity (e.g., heart rate, cortisol; (Ditzen et al., 2007) and less maladaptive emotion regulation (i.e., thought suppression; Debrot et al., 2014). Frequent kissing has been linked with lower perceived stress in daily life (Floyd et al., 2009), and more affectionate behaviors during marital interactions have been linked with higher relationship satisfaction (Carstensen et al., 1995). Being affectionately touched by one’s partner may further promote feelings of intimacy (Debrot et al., 2013), which in turn predicted more positive affect (Debrot et al., 2013); with some indication that individuals in satisfied relationships may derive more pronounced benefits from affectionate behaviors than those in dissatisfied relationships (Jakubiak, 2022).

Affection behavior has also been studied in dyadic interactions between married couples (Carstensen et al., 1995; Coan & Gottman, 2007). This research has revealed important differences in affection behavior when compared to other positive emotion behaviors (e.g., Carstensen et al., 1995). For example, in a longitudinal study on marital interaction (Verstaen et al., 2020), affection behavior was the only positive emotion that declined (for wives) or remained stable (for husbands) with age, while most other positive emotional behaviors (e.g., validation, humor) increased with age.

To the best of our knowledge, no study has examined subjective experiences of affection during dyadic interactions between spouses or friends. Addressing this gap is crucial before future studies can explore how experiences of affection may translate into affectionate behaviors (e.g., touch, hand-holding) during dyadic interaction.

**References**

Carstensen, L. L., Gottman, J. M., & Levenson, R. W. (1995). Emotional behavior in long-term marriage. *Psychology and aging*, *10*(1), 140–149. https://doi.org/10.1037/0882-7974.10.1.140

Coan, J. A., & Gottman, J. M. (2007). The Specific Affect Coding System (SPAFF). In J. A. Coan & J. J. B. Allen (Hrsg.), *Handbook of emotion elicitation and assessment* (S. 267–285). Oxford University Press.

Debrot, A., Schoebi, D., Perrez, M., & Horn, A. B. (2013). Touch as an interpersonal emotion regulation process in couples’ daily lives: The mediating role of psychological intimacy. *Personality and Social Psychology Bulletin*, *39*(10), 1373–1385. https://doi.org/10.1177/0146167213497592

Debrot, A., Schoebi, D., Perrez, M., & Horn, A. B. (2014). Stroking your beloved one’s white bear: Responsive touch by the romantic partner buffers the negative effect of thought suppression on daily mood. *Journal of Social and Clinical Psychology*, *33*(1), 75–97. https://doi.org/10.1521/jscp.2014.33.1.75

Ditzen, B., Neumann, I. D., Bodenmann, G., Von Dawans, B., Turner, R. A., Ehlert, U., & Heinrichs, M. (2007). Effects of different kinds of couple interaction on cortisol and heart rate responses to stress in women. *Psychoneuroendocrinology*, *32*(5), 565–574. https://doi.org/10.1016/j.psyneuen.2007.03.011

Floyd, K., Boren, J. P., Hannawa, A. F., Hesse, C., McEwan, B., & Veksler, A. E. (2009). Kissing in Marital and Cohabiting Relationships: Effects on Blood Lipids, Stress, and Relationship Satisfaction. *Western Journal of Communication*, *73*(2), 113–133. https://doi.org/10.1080/10570310902856071

Griffin, D., & Gonzalez, R. (1995). Correlational analysis of dyad-level data in the exchangeable case. *Psychological Bulletin*, *118*, 430–439. https://doi.org/10.1037/0033-2909.118.3.430

Jakubiak, B. K. (2022). Affectionate touch in satisfying and dissatisfying romantic relationships. *Journal of Social and Personal Relationships*, *39*(8), 2287–2315. https://doi.org/10.1177/02654075221077280

Jakubiak, B. K., & Feeney, B. C. (2017). Affectionate Touch to Promote Relational, Psychological, and Physical Well-Being in Adulthood: A Theoretical Model and Review of the Research. *Personality and Social Psychology Review*, *21*(3), 228–252. https://doi.org/10.1177/1088868316650307

O’Connor, B. P. (2019). *Package ‘WABA’* (1.0) [Software]. https://oconnor-psych.ok.ubc.ca/levels/WABA.pdf

Verstaen, A., Haase, C. M., Lwi, S. J., & Levenson, R. W. (2020). Age-related changes in emotional behavior: Evidence from a 13-year longitudinal study of long-term married couples. *Emotion*, *20*(2), 149–163. https://doi.org/10.1037/emo0000551

**Table S1**

*Means and Standard Deviations of Key Study Variables*

|  | Marriage Study | | | | Friendship Study | |
| --- | --- | --- | --- | --- | --- | --- |
| Variable | Husbands | | Wives | | Friends | |
|  | *M* | *SD* | *M* | *SD* | *M* | *SD* |
| Age | 43.62 | 8.52 | 42.18 | 8.81 | 19.98 | 2.22 |
| Relationship satisfaction | 90.67 | 25.35 | 92.61 | 26.95 | 3.45 | 0.87 |
| **Pleasant Conversation** | | | | | | |
| Affection | 5.80 | 2.07 | 6.16 | 2.05 | 5.62 | 2.21 |
| Amusement | 5.96 | 2.31 | 6.18 | 2.34 | 6.31 | 2.07 |
| Calm | 6.43 | 1.99 | 6.50 | 2.16 | 5.83 | 1.90 |
| Compassion | 5.57 | 2.31 | 5.59 | 2.20 | 5.54 | 2.02 |
| Excitement | 5.82 | 2.62 | 5.94 | 2.36 | 5.84 | 2.16 |
| Pride | 4.15 | 2.61 | 4.21 | 2.78 | 4.07 | 2.48 |
| Positive emotions (composite score without affection) | 5.60 | 1.68 | 5.72 | 1.59 | 5.52 | 1.50 |
| Anger | 1.57 | 1.19 | 1.80 | 1.87 | 1.35 | 1.11 |
| Disgust | 1.63 | 1.52 | 1.92 | 2.16 | 1.29 | 0.87 |
| Embarrassment | 1.51 | 0.87 | 1.65 | 1.44 | 1.75 | 1.25 |
| Fear | 1.73 | 1.47 | 1.71 | 1.60 | 1.39 | 0.86 |
| Sadness | 1.57 | 1.17 | 1.67 | 1.38 | 1.44 | 0.99 |
| Shame | 1.45 | 1.17 | 1.55 | 1.42 | 1.32 | 0.77 |
| Negative emotions (composite score) | 1.58 | 1.05 | 1.73 | 1.33 | 1.43 | 0.65 |
| **Conflict Conversation** | | | | | | |
| Affection | 4.82 | 2.60 | 4.86 | 2.35 | 4.93 | 2.30 |
| Amusement | 3.96 | 2.49 | 4.24 | 2.70 | 5.37 | 2.29 |
| Calm | 4.86 | 2.42 | 4.94 | 2.39 | 5.11 | 2.11 |
| Compassion | 4.53 | 2.51 | 5.29 | 2.46 | 5.44 | 2.11 |
| Excitement | 4.04 | 2.41 | 3.42 | 2.40 | 4.51 | 2.48 |
| Pride | 3.35 | 2.45 | 2.59 | 1.98 | 3.86 | 2.46 |
| Positive emotions (composite score without affection) | 4.15 | 1.83 | 4.11 | 1.69 | 4.85 | 1.70 |
| Anger | 3.78 | 2.36 | 3.14 | 2.50 | 1.88 | 1.41 |
| Disgust | 2.80 | 2.05 | 2.47 | 2.35 | 1.50 | 1.17 |
| Embarrassment | 3.10 | 2.38 | 2.94 | 2.34 | 2.94 | 1.92 |
| Fear | 2.73 | 2.36 | 2.31 | 1.98 | 2.18 | 1.69 |
| Sadness | 2.94 | 2.12 | 3.16 | 2.27 | 1.88 | 1.37 |
| Shame | 3.14 | 2.46 | 2.63 | 2.18 | 2.15 | 1.64 |
| Negative emotions (composite score) | 3.08 | 1.63 | 2.77 | 1.67 | 2.09 | 1.13 |

*Note. M* = Mean, *SD* = Standard deviation. Relationship satisfaction was measured using the Locke-Wallace Short Marital-Adjustment Test (Marriage Study; possible range: 2-158) and the McGill Friendship Questionnaire, satisfaction subscale (Friendship Study, possible range: -4 to 4).

**Table S2**

*Intercorrelations Between Key Study Variables (Marriage Study)*

| Variable | 1 | 2 | 3 | 4 | 5 | 6 | 7 | 8 | 9 |
| --- | --- | --- | --- | --- | --- | --- | --- | --- | --- |
| 1. Affection (pleasant conversation) | **.08** | .56^**^ | -.13 | .42^**^ | .34^*^ | .13 | -.14 | .01 | .06 |
| 2. Positive emotions (pleasant conversation) | .70^**^ | **.03** | .04 | .16 | .48^**^ | .29^*^ | -.30^*^ | .18 | -.40^**^ |
| 3. Negative emotions (pleasant conversation) | -.08 | -.02 | **.69^**^** | .03 | .06 | .29^*^ | -.04 | .05 | .10 |
| 4. Affection (conflict conversation) | .38^**^ | .35^*^ | -.03 | **.13** | .59^**^ | -.32^*^ | .02 | -.27 | -.05 |
| 5. Positive emotions (conflict conversation) | .20 | .36^*^ | -.03 | .72^**^ | **.02** | -.34^*^ | -.08 | -.13 | -.27 |
| 6. Negative emotions (conflict conversation) | -.03 | .04 | .68^**^ | -.22 | -.32^*^ | **.53^**^** | -.17 | .17 | .08 |
| 7. Relationship satisfaction | .38^**^ | .27 | -.20 | .25 | .31^*^ | -.24 | **.70^**^** | -.01 | .09 |
| 8. Age | .02 | .10 | -.16 | .07 | -.04 | -.11 | -.01 | **.84^**^** | -.11 |
| 9. SES | -.02 | -.17 | -.01 | -.32^*^ | -.48^**^ | .18 | -.11 | .08 | **.77^**^** |

*Note.* Positive emotions = composite score (amusement, calm, compassion, excitement, pride), negative emotions = composite score (anger, disgust, embarrassment, fear, sadness, shame). Wives’ correlations are below the diagonal; husbands’ correlations are above the diagonal; correlations between husbands and wives are on the diagonal in bold.

* *p* < .05., ** *p* < .01.

**Table S3**

*Intercorrelations Between Key Study Variables (Friendship Study)*

| Variable | 1. | 2. | 3. | 4. | 5. | 6. | 7. | 8. | 9. | 10. |
| --- | --- | --- | --- | --- | --- | --- | --- | --- | --- | --- |
| 1. Affection (pleasant conversation) | **.20^*^** |  |  |  |  |  |  |  |  |  |
| 2. Positive emotions (pleasant conversation) | .53^***^ | **-.04** |  |  |  |  |  |  |  |  |
| 3. Negative emotions (pleasant conversation) | .08 | .07 | **-.02** |  |  |  |  |  |  |  |
| 4. Affection (conflict conversation) | 0.70^***^ | 0.48^***^ | 0.11 | **.19** |  |  |  |  |  |  |
| 5. Positive emotions (conflict conversation) | 0.35^***^ | 0.68^***^ | 0.07 | .55^***^ | .**11** |  |  |  |  |  |
| 6. Negative emotions (conflict conversation) | 0.23^**^ | 0.18^**^ | 0.59^***^ | .06 | -.09 | **.26^**^** |  |  |  |  |
| 7. Relationship satisfaction | .11 | .13 | -.36^***^ | .11 | .09 | -.20^**^ | **.14** |  |  |  |
| 8. Age | .14 | .04 | .20^**^ | .16^*^ | .17^*^ | .12 | -.15^*^ | **.86^***^** |  |  |
| 9. SES (family) | .02 | -.08 | -.02 | .07 | -.04 | -.01 | .04 | -.08 | **.30^**^** |  |
| 10. SES (personal) | .05 | .03 | .04 | .10 | .16^*^ | -.03 | -.05 | .61^***^ | .06 | **.48^***^** |

*Note.* Positive emotions = composite score (amusement, calm, compassion, excitement, pride), negative emotions = composite score (anger, disgust, embarrassment, fear, sadness, shame). Presented are the overall within-partner correlations below the diagonal, and the correlations between friends are presented on the diagonal (bold) as intra-class correlations, according to Griffin & Gonzalez, (1995). P-values were adjusted for dependent observations in indistinguishable dyads (see Griffin & Gonzalez, 1995; O’Connor, 2019).

* *p* < .05., ** *p* < .01, ***, *p* < .001.
